# The carotid web: Current research status and imaging features

**DOI:** 10.3389/fnins.2023.1104212

**Published:** 2023-02-13

**Authors:** Shixiao Liang, Peixin Qin, Lili Xie, Shanshan Niu, Junqi Luo, Fei Chen, Xiangmeng Chen, Jie Zhang, Guojie Wang

**Affiliations:** ^1^Department of Radiology, The Fifth Affiliated Hospital of Sun Yat-sen University, Zhuhai, China; ^2^Kangda College of Nanjing Medical University, Lianyungang, China; ^3^Teaching Section, The Fifth Affiliated Hospital of Sun Yat-sen University, Zhuhai, China; ^4^Department of Ultrasound, The Fifth Affiliated Hospital of Sun Yat-sen University, Zhuhai, China; ^5^Department of Radiology, Jiangmen Central Hospital, Jiangmen, China; ^6^Department of Radiology, Zhuhai People’s Hospital, Zhuhai, China

**Keywords:** carotid web, ischemic stroke, imaging, CT, MRI, digital subtraction angiography (DSA)

## Abstract

The carotid web is commonly found in the carotid bulb or the beginning of the internal carotid artery. It presents as a thin layer of proliferative intimal tissue originating from the arterial wall and extending into the vessel lumen. A large body of research has proven that the carotid web is a risk factor for ischemic stroke. This review summarizes the current research status of the carotid web and focuses on its imaging presentation.

## 1. Introduction

The carotid web presents as a thin layer of proliferative intimal tissue originating from the arterial wall and extending into the lumen of the vessel. It is regarded as a high-risk factor for ischemic stroke of undetermined etiology (Kim et al., 2018; Mac Grory et al., 2021). When untreated, it predisposes patients to recurrent stroke, this risk of recurrent stroke is particularly high for young patients with cryptogenic stroke (Joux et al., 2014, 2016; Choi et al., 2015). Because of its low incidence, the carotid web has been mainly described by case reports and case series, resulting in its poor understanding by clinicians and radiologists. Therefore, it has a low detection rate and is often inaccurately diagnosed in clinical practice. This review summarizes the current knowledge of the carotid web and focuses on its imaging features.

## 2. Carotid web and ischemic stroke

### 2.1. Epidemiology

Rainer et al. (1968) first discovered the carotid web in 1968; thereafter, studies of the carotid web increased. During the following decades, carotid web research has developed, and its incidence in ischemic stroke is about 1.2% (Mac Grory et al., 2021), and it occurs at a higher incidence in males than females (Compagne et al., 2018; Mac Grory et al., 2021; El-Masri et al., 2023). Significantly more patients experience unilateral carotid webs compared to bilateral carotid webs. Furthermore, the reported number of black individuals with a carotid web is greater than that of individuals of other races; however, no large-scale epidemiological research on its incidence related to race has been reported (Mac Grory et al., 2020).

### 2.2. Pathology and pathogenesis

Pathologically, the carotid web presents as a specific type of fibromuscular dysplasia. One study of the pathology of the carotid web indicated that it was possible to observe the membrane that formed on the carotid artery wall. A longitudinal section showed vascular intimal hyperplasia accompanied by fibrosis and myxoid degeneration that protruded into the lumen to form a valve (Rodríguez-Castro et al., 2022). Furthermore, a review that retrospectively analyzed the pathological results of 21 cases of carotid web found abnormalities in the intimal artery layer (Kim et al., 2018). Based on these results, the carotid web mainly involves the intimal layer, which is in contrast to the media’s major involvement in myofibers’ typical dysplasia.

The pathogenesis of the carotid web has not yet been clarified. It has been suggested that the carotid web is a congenital abnormality caused by genetic factors or a disorder associated with vascular injury or abnormal hormone levels *in vivo* (Olin et al., 2014). Moreover, because some oral contraceptives can lead to hyperplasia of the intimal artery layer, the carotid web in young females might be attributable to the use of contraceptives (Irey et al., 1978).

### 2.3. Carotid web and ischemic stroke

Coutinho et al. (2017) found that the carotid web incidence was 9.4% (5/53) for patients with ischemic stroke of undetermined cause; however, it decreased to 1% (1/102) in another cohort including age-matched and sex-matched patients who underwent computed tomography angiography (CTA) for other cerebrovascular diseases (the difference between the two cohorts was statistically significant). Compelling evidence has been reported by Sajedi et al. (2017) and Haussen et al. (2018), whom both indicated a certain relationship between the carotid web and ischemic stroke.

Currently, the mechanism by which the carotid web induces ischemic stroke is debatable. It is presumed that the aberrant protrusion of proliferative myofibers into the vessel lumen disturbs the balance of intraluminal flow, thus causing the flow velocity to be slow or turbulent and promoting the formation of a mural thrombus, eventually leading to stroke or stroke recurrence (Choi et al., 2015). One review indicated that 12–29% of stroke patients with a carotid web had overlapping thrombosis (Kim et al., 2018). These findings may prove that the carotid web leads to local blood flow stagnation, followed by the development of thrombosis, thrombus fragmentation, and, finally, ischemic stroke (Choi et al., 2015).

Surgical procedures for the carotid web mainly include carotid endarterectomy and artery stenting, both of which have satisfactory outcomes (Joux et al., 2014; Lenck et al., 2014; Choi et al., 2015; Haussen et al., 2018; Marnat et al., 2022; Patel et al., 2022; Ren et al., 2022). Research has revealed that, during follow-up, surgically treated carotid web patients reported that they did not experience recurrent strokes (Joux et al., 2014; Choi et al., 2015). Drug therapies include anticoagulation and antiplatelet therapies (Joux et al., 2014; Choi et al., 2015). However, single antiplatelet therapy may not be sufficient to prevent recurrent ischemic stroke attributable to artery embolization resulting from the formation and dislodgement of thrombi caused by flow turbulence and stasis after carotid web occurrence. Researchers found that 30% of patients who received antiplatelet therapy only suffered recurrent ischemic strokes, the median time of recurrence was 12 months, the earliest time was 1 month, and the other time was equal to or greater than 6 months (Joux et al., 2014). In light of the fact blood stasis can be the major cause of thrombosis, and that antiplatelet aggregation drugs have a limited role in blood stasis, it may be more appropriate to use anticoagulation therapy to prevent ischemic strokes caused by the carotid web.

## 3. Imaging features

Current imaging techniques for the diagnosis of carotid web are mainly digital subtraction angiography (DSA), CTA, magnetic resonance imaging (MRI), and ultrasound.

### 3.1. Digital subtraction angiography

**FIGURE 1:**
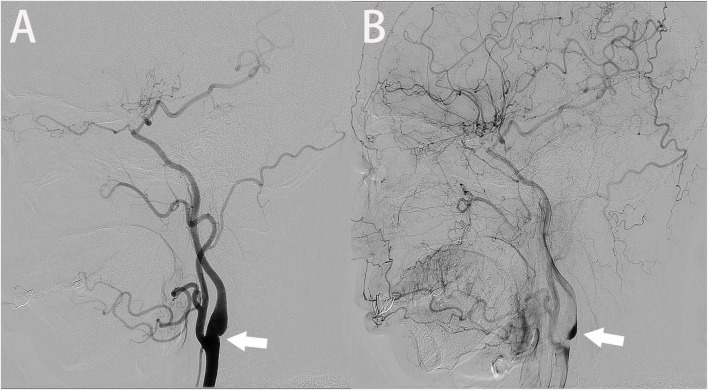
A digital subtraction angiography image shows a filling defect in the posterior wall of the carotid bulb (**A**, arrow). The venous phase image shows continued contrast pooling in the distal part of the carotid web (**B**, arrow).

Digital subtraction angiography was first used for the diagnosis of the carotid web with a linear filling defect along the carotid artery wall or a shelf-like filling defect located in the internal carotid artery bulb (Figure 1A). During the late venous phase, contrast agent retention was observed at the distal end of the carotid web (Figure 1B). Compared with atherosclerosis, the carotid web showed a lesser degree of stenosis on DSA (Park et al., 2022). Despite its reliability and safety, DSA remains an invasive technique. Additionally, since the carotid web is located mainly on the posterior wall of the internal carotid artery, and the internal carotid artery is located in the posterolateral direction, misdiagnosis may result if DSA obtains only two standard projections, front and side (Lenck et al., 2014).

### 3.2. Computed tomography angiography

**FIGURE 2:**
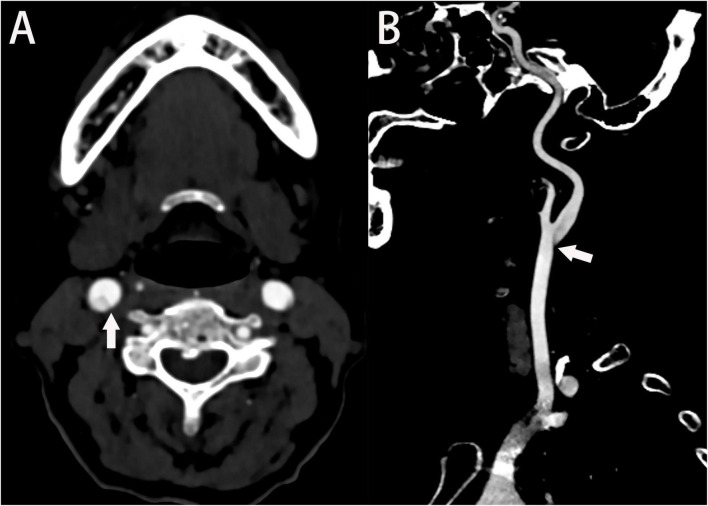
Axial **(A)** and sagittal **(B)** computed tomography angiography images of the neck show thin, shelf-like filling defects in the posterolateral wall of the carotid bulb (arrows).

**FIGURE 3:**
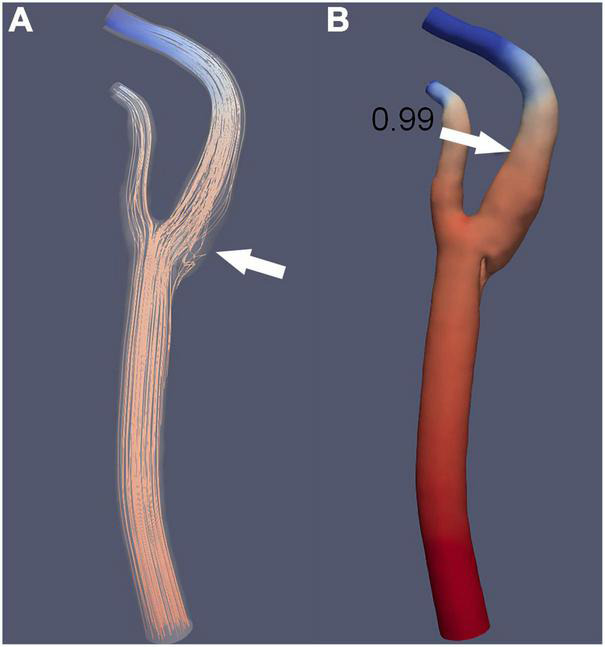
A simulation diagram of computational fluid dynamics reveals an extensive recirculation zone at the distal end of the carotid web (**A**, arrow); however, there is no decrease in the fractional flow reserve (**B**, arrow).

Carotid CTA has multiple advantages, such as its high resolution, short scan duration, and capability to construct arbitrary azimuth images. It can construct related vessels quickly. Typically, a low-density shadow with the appearance of a thin line can be seen in the transverse axial plane (Figure 2A), and the sagittal plane reveals a localized membrane-filling defect (Figure 2B; Choi et al., 2015). In clinical practice, carotid web can only be diagnosed with CTA if the two conditions are simultaneously present. The presence of only one of these two manifestations may lead to a missed diagnosis or misdiagnosis because it is difficult to distinguish the carotid web from carotid atherosclerotic plaque and other disorders. CTA can construct images at different angles, thereby allowing for the differentiation of carotid web from other diseases, such as carotid dissection and aneurysm. Based on the density difference, CTA can reveal whether thrombosis occurs in the carotid web (Wang et al., 2021). A previous report showed that CTA and DSA are associated with similar carotid web detection rates (Madaelil et al., 2019). CTA is relatively inexpensive, easy to perform, and safe compared to magnetic resonance angiography (MRA) and DSA. During a study performed to clarify the relationship between the carotid web and ischemic stroke, the length, area, and volume of the carotid web were measured using CTA images of both symptomatic and asymptomatic cases (Perry da Camara et al., 2022), resulting in the following observations among symptomatic and asymptomatic cases, respectively: mean carotid web lengths of 3.2 and 2.5 mm; median areas of 5.8 and 5.0 mm^2^; and median volumes of 15.0 and 10.6 mm^3^. Additionally, that study noted that patients with a thinner carotid web (the longer intraluminal projection compared with the base) were more likely to have symptoms. Another study assessed the hemodynamic parameters of the carotid web using CTA images of patients with confirmed carotid webs (Compagne et al., 2019). The results showed intravascular flow disturbances in the carotid web that might lead to dysfunction of the vessel wall and increase the likelihood of platelets adhering to each other and coagulating, eventually resulting in thrombosis of the distal carotid web. Combining CTA with artificial intelligence to assess hemodynamic changes induced by the carotid web is a novel technique that should be researched. A simulation diagram of computational fluid dynamics is shown in Figure 3.

### 3.3. Magnetic resonance imaging

**FIGURE 4:**
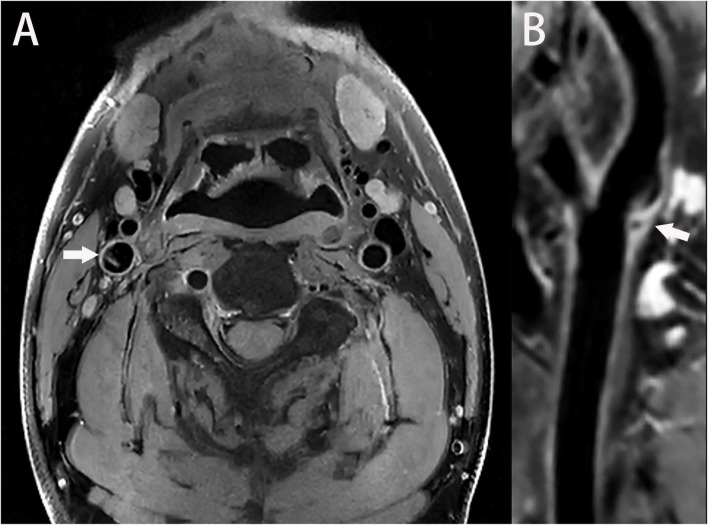
**(A)** A high-resolution magnetic resonance T1-weighted imaging (T1WI) axial image shows a thin, membrane-like structure protruding from the wall of the internal carotid artery to the lumen; furthermore, this structure separates the lumen (arrow). **(B)** A T1WI-enhanced sagittal image shows a locally enhanced, high signal on the carotid web (arrow).

Magnetic resonance angiography is a common MRI modality used to diagnose the carotid web. On MRA, the carotid web mainly presents as a film-like structure originating from the posterior carotid artery wall and protruding into the vessel lumen. In most cases, the carotid web appears upward and inward on MRA, and its morphology is consistent with that on CTA (Lantos et al., 2016; Boesen et al., 2017). Using a two-dimensional fast spin echo sequence and two-dimensional cine fast spin echo, researchers found a thickened vessel wall with the carotid web and increased signal enhancement (Boesen et al., 2017). Additionally, normal pulsatile expansion of the unilateral vessel wall, which was different from the homogeneous expansion of the healthy carotid artery, was observed (Boesen et al., 2017). Vessel wall MRI is an imaging technique used to obtain vessel wall images with blood suppression. According to a previous case report, vessel wall MRI was used to observe a carotid web patient, and a film-like protrusion in the vessel wall presented with isointense on T1-weighted imaging, a slightly high signal on fat-suppressed T2-weighted imaging, and significant enhancement on an enhanced scan was observed (Gopal et al., 2020). Moreover, because of its multiparameter and multisequence features, vessel wall MRI allows the multidirectional structural characterization of various vessel wall disorders (Figure 4; Oppenheim et al., 2009).

Magnetic resonance imaging provides carotid web-related information such as the composition of the vessel wall, hemodynamics, and peripheral blood. Moreover, it can accurately assess cerebral infarctions; therefore, it is conducive to a more comprehensive evaluation of the carotid web. Additionally, Phase Contrast MRA and Time of Flight-MRA can be alternatives to CTA for patients who are pregnant or have renal insufficiency because they are radiation-free and without contrast administration. Contrast Enhanced MRA also requires contrast agent injection but displays more vascular information. In contrast to CTA, Enhancement MRA has the disadvantage of taking a significant amount of time to scan and post-process. Nonetheless, the diagnosis of the carotid web with MRI has been poorly studied, and there have been few studies of MRI sequences for diagnosing the carotid web. Therefore, further studies are required to confirm these results.

### 3.4. Ultrasound

**FIGURE 5:**
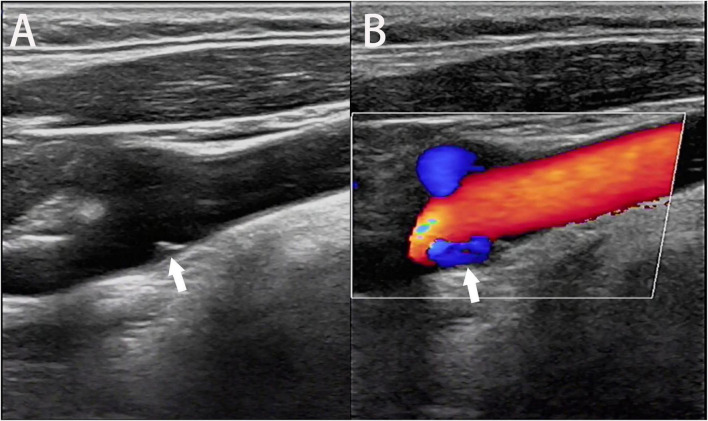
Ultrasonic image of the longitudinal section shows the carotid web extending into the lumen of the carotid bulb (**A**, arrow). An eddy current is generated at the angle between the carotid web and the artery wall on the color Doppler flow image (**B**, arrow).

During routine ultrasound, the carotid web presents as a film-like structure that is isoechoic or hypoechoic and originates from the vessel wall of the carotid artery and protrudes into the lumen with or without peripheral atherosclerosis (Fontaine et al., 2022; Figure 5A). Additionally, it generally presents with “cliff-like” arterial stenosis in the longitudinal section (Ben et al., 2022), which is superior to the manifestation in the transverse section in terms of the carotid web diagnosis. Using the color Doppler, an eddy can be found at the angle between the carotid web and vessel wall (Phair et al., 2017; Figure 5B). Routine ultrasound performed to diagnose the carotid web is convenient and safe; however, it may lead to misdiagnoses, including carotid dissection and ulcerative plaque. Therefore, multiple techniques, such as three-dimensional ultrasound imaging, contrast-enhanced ultrasonography, and intravascular ultrasound, have been developed as alternatives to routine ultrasound to reduce the misdiagnosis rate. Three-dimensional ultrasound imaging can depict the anatomical structure of the carotid web using three-dimensional multiplanar reconstruction (Gao et al., 2022). Contrast agent infusion delays and filling defects were observed using contrast-enhanced ultrasonography (Zhou et al., 2022). Intravascular ultrasound provides a dynamic view of the vessel lumen using an ultrasonic mini-probe with a catheter technique, and the carotid web presents with a banding hypoechoic signal connecting to the vessel wall (Hassani et al., 2020).

In contrast, ultrasound is less expensive and more convenient than other imaging examinations, is non-invasive, and can be used as an auxiliary method to evaluate the carotid web.

There is no clear consensus regarding the best imaging method for carotid webs. Research on several imaging methods, including MRI, is lacking. Therefore, further research on the relationship between stroke and the carotid web should include but is not limited to, the use of CT or MRI to assess intracranial infarcts and perfusion and their relationship with carotid web features, such as size and angle, and the application of artificial intelligence to evaluate hemodynamic changes in the carotid web.

## 4. Summary

The carotid web will gain more attention as its research progresses. However, there are still many areas in the current research that can be supplemented, such as the pathogenesis of the carotid web, which remains unclear, the correlation between the carotid web and ischemic stroke, which requires more standardized data for further analysis, and the carotid web intervention and treatment strategy choices. CTA has been the recommended method for the diagnosis of the carotid web in most studies; however, other imaging methods have been proven to be very good for diagnosing and evaluating the carotid web. Therefore, multiple imaging modalities combined would provide more information on the carotid web.

## Author contributions

SL: writing—original draft and investigation. PQ: methodology, investigation, and writing—original draft. LX: conceptualization, investigation, and writing—original draft. SN: validation and visualization. JL: validation. FC: methodology. XC: writing—review and editing, funding acquisition, resources, and visualization. JZ: visualization, writing—review and editing, and resources. GW: conceptualization, writing—original draft, writing—review and editing, funding acquisition, visualization, and resources. All authors contributed to the article and approved the submitted version.
